# The choice of extraction site modulates the incidence of incisional hernia in colorectal surgery: a cohort analysis

**DOI:** 10.1007/s13304-025-02272-4

**Published:** 2025-06-27

**Authors:** Marie Burgard, Emilie Liot, Guillaume Meurette, Pierre-Alexandre Poletti, Christian Toso, Frédéric Ris, Jeremy Meyer

**Affiliations:** 1 Division of Digestive Surgery, University Hospitals of Geneva, Rue Gabrielle-Perret-Gentil 4, 1205 Geneva, Switzerland; 2 Medical School, University of Geneva, Geneva, Switzerland; 3 Division of Radiology, University Hospitals of Geneva, Geneva, Switzerland

**Keywords:** Incisional hernia, Colectomy, Minimally invasive surgical procedures, Extraction site

## Abstract

Although minimally invasive surgery has significantly reduced the incidence of incisional hernia (IH) in colorectal procedures, the choice of specimen extraction site continues to pose a risk. This study explores how the location of extraction site influences the occurrence of IH in patients undergoing minimally invasive colorectal resections. We conducted a retrospective observational cohort study involving consecutive patients who underwent minimally invasive colorectal resection for colorectal cancer from 2013 to 2021. Patients with open surgery, previous hernia repair, reoperations during the study period or without imaging follow-up were excluded. The primary outcome measured was the CT-proven incidence of IH at the extraction site. A total of 191 patients were analyzed. Among them, 113 patients (59.2%) had a midline extraction site, while and 78 patients (40.8%) had an off-midline extraction. Midline extraction was preferably used during right and transverse colectomy (98% and 100% respectively), whereas off-midline were preferred in left hemicolectomy, sigmoidectomy, and anterior resection (55%, 88%, 95% respectively). Remarkably, the overall incidence of IH at the extraction site was 30.9% for midline extractions compared to 0% for off-midline extractions (*p* value < 0.001). The mean follow-up duration was of 3.3 ± 2.1 years. Multivariate logistic regression analysis revealed midline extraction as a significant risk factor for IH with a relative risk of 29.1 (95% CI 3.8–220.5, *p* value < 0.001). The findings highlight a substantial increase in the risk of IH associated with midline extraction sites. As such, it is crucial to advocate for fully minimally invasive colorectal resection using off-midline incision to enhance patient outcomes and reduce the risk of incisional hernias.

**Supplementary Information:**

The online version contains supplementary material available at 10.1007/s13304-025-02272-4.

## Introduction

Minimally invasive techniques have revolutionized colorectal surgery, offering significant benefits such as reduced hospital stays and decreased post-operative pain while maintaining comparable oncologic outcomes [1–7]. However, concerns remain regarding the incidence of incisional hernia (IH) following these procedures [8–10].

The choice of specimen extraction site, midline or off-midline is often influenced by surgeons’ preference, resection type and anatomic considerations. Besides the extraction of the specimen, the incision can be used to perform extracorporeal lymphadenectomy and anastomosis, in right hemicolectomies for instance, the midline incision is often preferred.

The choice of the extraction site is known, however, to modulate the overall incidence of IH, and particularly the incidence of IH at the extraction site [9, 11, 12]. The midline incision has been identified to be at higher risk for IH than off-midline incisions [11, 13–15]^.^ IH represent a significant burden for healthcare [16], requiring additional surgical procedures [17], emergency admissions and by affecting patients’ quality of life [18]. This study aims to Investigate the relationship between specimen extraction site choice and the development of IH at the extraction site after laparoscopic elective colorectal surgery for cancer.

## Methods

### Study design

Data of all consecutive patients who underwent elective laparoscopic colorectal surgery for colorectal cancer in our institution between January 2013 and December 2021 were retrospectively analyzed. Data were extracted from our in-hospital database. Collected data were patients demographics [age, sex, BMI (body mass index)], comorbidities, previous surgeries, immunosuppressive therapy, tobacco use, type of colonic resection, choice of specimen extraction site, duration of operation, length of stay, short-term post-operative complications and incidence of IH on long-term follow-up (minimum 1 year).

The exclusion criteria were a pre-existing ventral hernia, a previous ventral hernia repair, conversion to open surgery, extraction through the stoma site, reoperations during the study period or patients without an available follow-up computed tomography (CT) scan.

The primary outcome was the incidence of IH during the follow-up period (last follow-up: December 2023). IH was defined as a hernia occurring at the specimen extraction site on follow-up abdominal CT scan with a minimal follow-up of 1 year. All CT scans were independently reviewed by both, a radiologist and a digestive surgeon.

### Statistical analysis

The Statistical Package for Social Sciences, version 29.0 (SPSS, IBM, Armonk, USA.) for statistical analysis was used. Quantitative variables were expressed as median or mean ± standard deviation (SD) and range. Qualitative variables were expressed as raw numbers, proportions, and percentages. The Pearson’s Chi-square test was used to evaluate the difference between categorical variables, while the Student’s *t* test was used for continuous variables. Uni-and multivariate analysis with logistic regression were performed. The data were expressed as relative risks (RRs) with 95% confidence intervals. The Kaplan–Meyer method for assessing the cumulative incidence of IH during the follow-up was used.

The study was approved by the local ethics committee (ID 2023-0016).

## Results

Among 604 eligible patients who benefited from minimally invasive colorectal resection for colorectal cancer, 191 were included in the analysis, 316 patients had to be excluded because of a missing follow-up CT scan and 97 patients met other exclusion criteria.

**Fig. 1:**
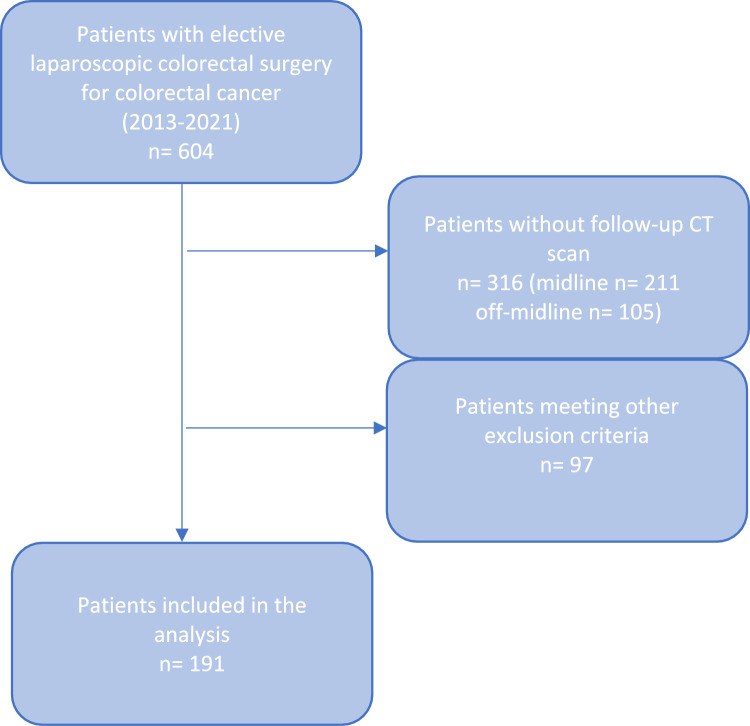
Inclusion flow-chart

The mean follow-up was 3.3 ± 2.1 years, Fig. 1 summarizes the inclusion flow-chart.

One hundred thirteen patients (59.2%) had an extraction site located on the midline, and 78 patients (40.8%) had an off-midline extraction site. There was no significant difference in the median age (67 years vs 63 years, *p* = 0.062) or the mean BMI (26.5 kg/m^2^ vs 26.3 kg/m^2^, *p* = 0.866) between patients of both groups. There were significantly more male patients in the off-midline group (65% vs 46%, *p* = 0.030). Comorbidities, tobacco use, or immunosuppressive medications were similar between both groups.

The operative time was significantly shorter (− 33 min, *p* value = 0.002) and the hospital stay was significantly longer in the midline group (+ 2.1 days, *p* value = 0.004). The midline was preferably used as extraction site during right and transverse colectomies (98% and 100%, respectively), and off midline was used in left hemicolectomy, sigmoid colectomy, and anterior resection (55%, 88%, 95%, respectively). Patients’ demographics and peri-and post-operative data are summarized in Table 1.

**Table 1:** Patients’ demographics, peri-and post-operative data

	Total *N* = 191	Midline *N* = 113	Off-midline *N* = 78	*p*
Age, years, median (range)	65 (17–89)	67 (17–89)	63 (42–87)	0.042
Sex, *n* (%)				
F	88 (46.1)	61 (54)	27 (34.6)	0.030
H	103 (53.9)	52 (46)	51 (65.4)	
BMI, kg/m^2^, mean (SD)	26.4 (± 5.3)	26.5 (± 5.3)	26.3 (± 5.2)	0.866
Comorbidities, *n* (%)				
Hypertension	82 (42.9)	52 (47.3)	30 (38.5)	0.906
Cardiopathy	23 (12)	15 (13.6)	8 (10.3)	
Diabetes	30 (15.7)	18 (16.4)	12 (15.4)	
COPD	16 (8.4)	11 (10)	5 (6.4)	
Tabaco				
Actif	44 (23)	25 (23.6)	18 (23,4)	
Past	25 (13.1)	12 (10.9)	13 (16.9)	0.665
Immunosuppressive medication	2 (1)	1 (0.9)	1 (1.3)	0.665
Type of résection, *n* (%)				
Right hemicolectomy	93 (48.7)	91 (80.5)	2 (2.6)	
Left hemicolectomy	20 (10.5)	9 (8)	11 (14.1)	0.001
Transverse colectomy	7 (3.7)	7 (6.2)	0	
Sigmoidectomy	25 (13.1)	3 (2.7)	22 (28.2)	
Rectal resection	43 (22.5)	2 (1.8)	41 (52.6)	
Total/subtotal colectomy	3 (1.6)	1 (0.9)	2 (2.6)	
Operative time, minutes, mean (SD)	213.3 (± 70.2)	191 (± 58.7)	224 (± 68.2)	0.002
Post-operative complications, n (%)	36 (18.8)	22 (21.2)	5 (9.3)	
Ileus	17	14	1	0.075
Other	18	8	4	
Hospital stay, days, mean (SD)	8.6 (± 4.9)	9.4 (± 5.3)	7.3 (± 4.2)	0.004

The standard abdominal wall closure technique in midline and off-midline incisions was small stitches (5 mm bite size, 5-mm step size) with slowly absorbable monofilament suture.

Midline extraction sites included peri-umbilical incisions (94/113 patients, 83%), supra-umbilical incisions (6/113 patients, 5%) and infra-umbilical incisions (8/113 patients, 7%). Off- midline extraction sites included Pfannenstiel incisions (77/78 patients, 99%) and transverse incisions (1/78 patients, 1.3%).

**Fig. 2:**
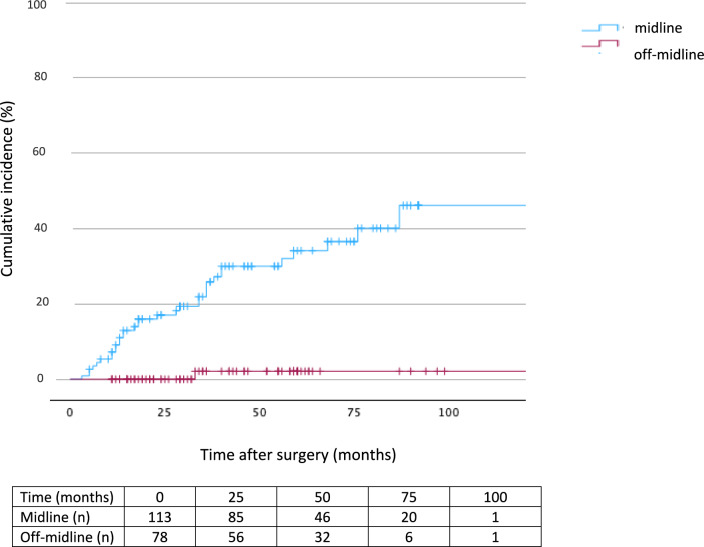
Incidence of incisional hernia at the level of the extraction site, midline, and off -midline

The overall incidence of IH at the level of the extraction site was 30.9% (35 patients) when midline was chosen as extraction site and 0% (no patient) when the extraction site was off-midline (*p* value < 0.001).There was no statistical difference in the mean follow-up (3.4 ± 2.1 years vs 3.1 ± 2.1 years, *p* = 0.279) or the frequency of CT scans (1.2 (± 1.1) CT scans/years vs 1.4 ± 1.1 CT scans/year, *p* = 0.078) between groups. When the midline was chosen as an extraction site, IH was present in 30/94 patients (31.9%) with a peri-umbilical incision, 2/6 patient (33.3%) with a supra-umbilical incision and 3/8 (37.5%) patients with an infra-umbilical incision. When the extraction site was off-midline, IH was present in 0/78 patients (0%) with a Pfannenstiel incision and 0/1 patients (0%) with a transverse incision. The cumulative incidence of IH is illustrated in Fig. 2. Eight patients (7.1%) required hernia repair in the midline group and no patient (0%) in the off-midline group (*p* value = 0.051).

Table 2 shows the analysis of factors associated with IH on the specimen extraction site where stoma site extraction is excluded from the analysis. Uni-and multivariate logistic regression identified the choice of midline as an extraction site as a risk factor for IH (RR 29.1, 95% CI 3.8–220.5, *p* value < 0.001.

**Table 2:** Factors associated with incisional hernia on the specimen extraction site

	Incisional hernia *N* = 35	No incisional hernia *N* = 156	Monovariate *p* value	Multivariate RR (95% CI)	Multivariate *p* value
Age, years, median (range)	67.5 (19–88)	64.5 (17–89)	0.274	1.03 (0.97–1.03)	0.857
Gender, *n* (%)					
F	21 (60)	67 (42.9)	0.067	1.46 (0.63–3.4)	0.359
H	14 (40)	89 (86.4)			
BMI, kg/m^2^, mean (SD)	27.1 (± 5.5)	26.3 (± 5.3)	0.449	1.03 (0.96–1.16)	0.433
Comorbidities, *n* (%)	23 (69.7)	89 (57.4)	0.192	0.46 (0.16–1.4)	0.284
Tabaco use	11 (31.4)	60 (38.5)	0.727	0.98 (0.56–1.7)	0.929
Previous surgery	17 (50)	46 (49)	0.214	0.71 (0.31–1.92)	0.254
Operative time, minutes, mean (SD)	199 (± 60.7)	216 (± 72.6)	0.185	1.0 (0.99–1.0)	0.736
Extraction site, *n* (%)					
Midline	35 (100)	78 (50)	< 0.001	29.1 (3.8–220.5)	< 0.001
Peri-umbilical	30	64			
Supra-umbilical	2	4			
Infra-umbilical	3	5			
Off-midline	0 (0%)	78 (50)			
Pfannenstiel	0	77			
Transverse	0	1			
Post-operative complications, *n* (%)	10 (28.6)	26 (16.%)	0.104	0.7 (0.23–2.3)	0.553
Hospital stay, mean (SD)	9.9 (± 5.7)	8.3 (± 4.7)	0.080	1.03 (0.95–1.13)	0.490

*N* number, *F* female, *M* male, *BMI* body mass index, *SD* standard deviation, *RR* relative risk

## Discussion and conclusions

Our study shows that the midline specimen extraction site is an independent risk factor for the development of an extraction site IH in elective laparoscopic colorectal surgery for colorectal cancer. In our cohort, the risk for IH was 29-fold when the midline was chosen compared to an off-midline incision. These results are consistent with those of previously published studies [12, 15]. Moreover, the European Hernia society guideline on the closure of abdominal wall incisions recommend using a non-midline approach, whenever possible [19].

Increased BMI and surgical site infections are other known risk factors for incisional hernias [20, 21]. In our analysis, post-operative complications, and BMI did not account for the development of incisional hernias and the difference in hernia development was solely explained by the choice of extraction site.

Interestingly, patients with a Pfannenstiel incision had no IH during the follow-up. Other published papers in gynecologic or visceral surgery patient cohorts also show that the Pfannenstiel incision has the lowest incidence of IH compared to midline and other off-midline incisions [15, 22, 23].

In our cohort the midline extraction was preferably chosen when performing right or transverse hemicolectomy while the Pfannenstiel or transverse incisions were used for left hemicolectomy, sigmoid colectomy, or anterior resection. This is consistent with the common practice of most colorectal surgeons and is mainly explained by the difference in anastomotic techniques [11, 24]. In right and transverse colectomies, the anastomosis is mostly performed extracorporeally [25] and the extraction site also serves as access for the anastomosis whereas intracorporeal anastomosis are performed for left and sigmoid colectomies as well as rectal resections. In case of an extracorporeal anastomosis the midline incision gives better exposure than a transverse or Pfannenstiel incision. Moreover, multiple studies have shown a reduced incidence of IH in right colectomies or ileo-caecal resections with intracorporeal anastomosis when compared to extracorporeal anastomosis [26, 27]. However, intracorporeal anastomosis for laparoscopic right hemicolectomies can be challenging. The use of a robotic platform could be advantageous as surgeons more conveniently perform intracorporeal anastomosis with the robotic platform than laparoscopically [28, 29].

The high incidence of IH in the midline extraction group in our patient cohort (30%), might be explained by the diagnostic tool and the duration of follow-up. The incidence of IH is known to increase with increasing lengths of follow-up [19] and Fink et al. describe a 60% increase of IH rate from 1 to 3 years post-operative follow-up [30]. In addition, abdominal CT scan is the most sensitive examination modality for diagnosing IH [19, 31] and leads to a higher detection of IH than clinical examination. A significant proportion of these IH might not be clinically relevant. Indeed, in our patient cohort, only a small proportion of patients diagnosed with an IH underwent hernia repair.

The main limitation of our study is its retrospective character with its consequential limitations. We could not analyse the influence of the technique of abdominal wall closure on the development of IH and some of the post-operative complications, for instance wound infection, could be missing. Also, patients in whom we could not retrieve oncologic follow-up CT scan were not included in the analysis. Furthermore, patients with late-term complications, for instance IH, might have had a longer follow-up in a hospital setting than patients with an uncomplicated follow-up, possibly leading to an overestimation of the incidence.

Despite of these limitations we can conclude that choosing the midline as an extraction site exposes patients to the risk of IH. Fully minimally invasive colorectal resection using off-midline incision as extraction site should be encouraged.

## Supplementary Information

Below is the link to the electronic supplementary material.

Supplementary file1 (PNG 358 kb)

## Data Availability

The data that support the findings of this study are available on request from the corresponding author, [MB]. The data are not publicly available as they contain information that could compromise the privacy of research participants.
