# Supplementary figures and images for: The choice of extraction site modulates the incidence of incisional hernia in colorectal surgery: a cohort analysis
Source: Updates Surg. 2025 Jun 27;78(4):1581–7. doi: 10.1007/s13304-025-02272-4 (PMC13421187; doi:10.1007/s13304-025-02272-4)

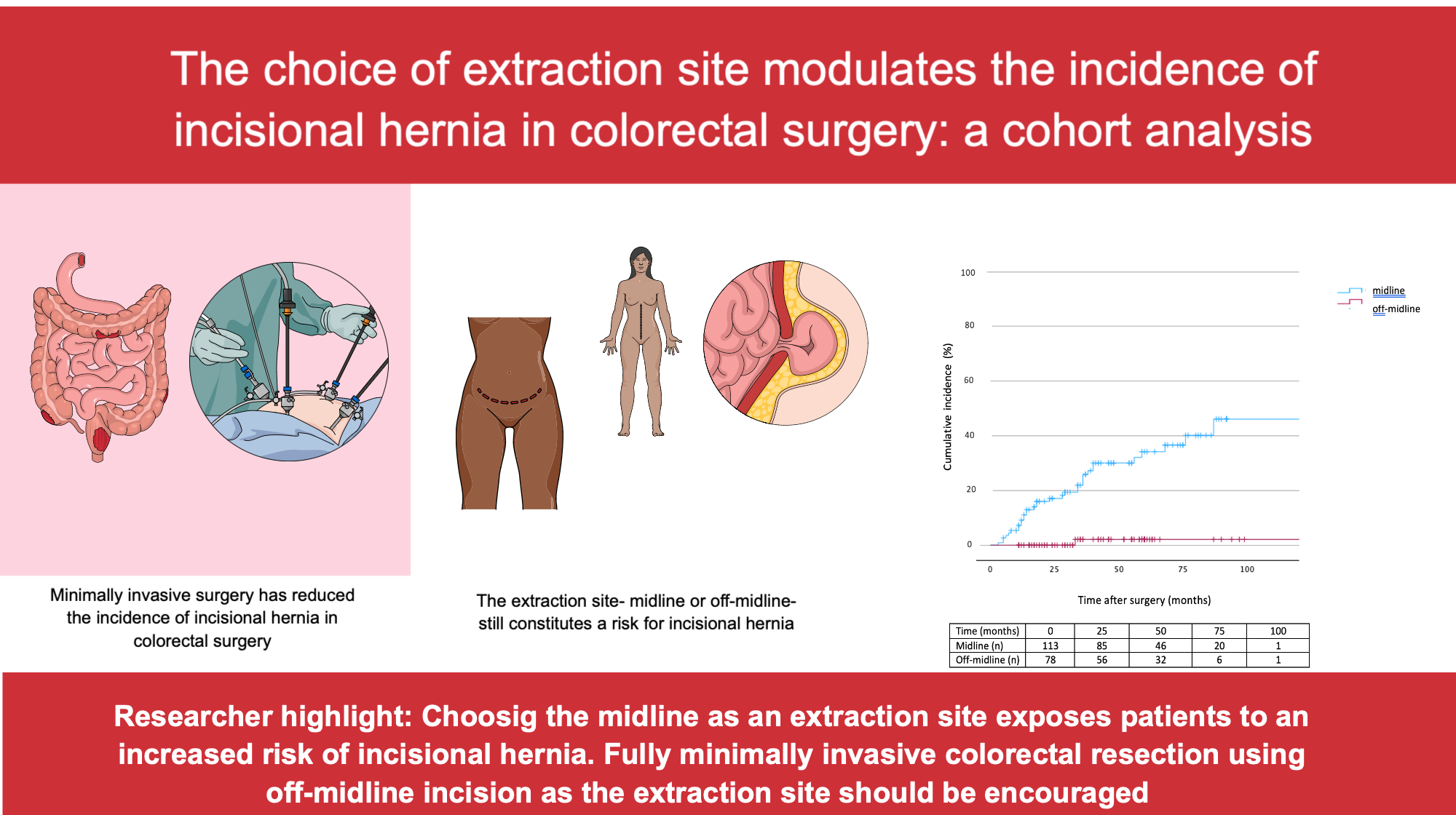

Supplement: Supplementary file 1 — Supplementary file1 (PNG 358 kb) [file 13304_2025_2272_MOESM1_ESM.png]
